# Costimulatory molecule expression profile as a biomarker to predict prognosis and chemotherapy response for patients with small cell lung cancer

**DOI:** 10.1007/s00262-022-03280-8

**Published:** 2022-08-24

**Authors:** Peng Wu, Zhihui Zhang, Zhaoyang Yang, Chaoqi Zhang, Yuejun Luo, Guochao Zhang, Lide Wang, Qi Xue, Nan Sun, Jie He

**Affiliations:** 1 Department of Thoracic Surgery, National Cancer Center/National Clinical Research Center for Cancer/Cancer Hospital, Chinese Academy of Medical Sciences and Peking Union Medical College, Beijing, 100021 China; 2 State Key Laboratory of Molecular Oncology, National Cancer Center/National Clinical Research Center for Cancer/Cancer Hospital, Chinese Academy of Medical Sciences and Peking Union Medical College, Beijing, 100021 China; 3 Department of Pathology, National Cancer Center/ National Clinical Research Center for Cancer/Cancer Hospital,, Chinese Academy of Medical Sciences and Peking Union Medical College, Beijing, 100021 China

**Keywords:** Costimulatory molecule, Prognostic biomarker, Chemotherapy response, Small cell lung cancer, Individualized medicine

## Abstract

Owing to the paucity of specimens, progress in identifying prognostic and therapeutic biomarkers for small cell lung cancer (SCLC) has been stagnant for decades. Considering that the costimulatory molecules are essential elements in modulating immune responses and determining therapeutic response, we systematically revealed the expression landscape and identified a costimulatory molecule-based signature (CMS) to predict prognosis and chemotherapy response for SCLCs for the first time. We found T cell activation was restrained in SCLCs, and costimulatory molecules exhibited widespread abnormal genetic alterations and expression. Using a LASSO Cox regression model, the CMS was built with a training cohort of 77 cases, which successfully divided patients into high- or low-risk groups with significantly different prognosis and chemotherapy benefit (both *P* < 0.001). The CMS was well validated in an independent cohort containing 131 samples with qPCR data. ROC and C-index analysis confirmed the superior predictive performance of the CMS in comparison with other clinicopathological parameters from different cohorts. Importantly, the CMS was confirmed as a significantly independent prognosticator for clinical outcomes and chemotherapy response in SCLCs through multivariate Cox analysis. Further analysis revealed that low-risk patients were characteristic by an activated immune phenotype with distinct expression of immune checkpoints. In summary, we firstly uncovered the expression heterogeneity of costimulatory molecules in SCLC and successfully constructed a novel predictive CMS. The identified signature contributed to more accurate patient stratification and provided robust prognostic value in estimating survival and the clinical response to chemotherapy, allowing optimization of treatment and prognosis management for patients with SCLC.

**Supplementary Information:**

The online version contains supplementary material available at 10.1007/s00262-022-03280-8.

## Introduction

Lung cancer is an aggressive malignancy worldwide, with 2.2 million new cases and 1.8 million deaths estimated in 2020, contributing to almost 20% of cancer-related deaths [1]. Small cell lung cancer (SCLC), which accounts for approximately 15% of all lung cancers [2], represents a highly lethal subtype with the characteristics of concealed pathogenesis, rapid growth, and early metastasis, which pose great challenges to efforts aimed at improving the poor prognosis and high mortality associated with this type of cancer [3]. The standard treatment strategy of SCLC, which includes chemotherapy combined with radiotherapy, has remained unchanged for more than three decades [4]. Although SCLC patients generally show a good response to the initial treatment, most cases rapidly develop drug resistance or relapse after remission [5]. Therefore, it is imperative to explore effective biomarkers for prognostic and therapeutic prediction, as well as elucidate the mechanisms underlying metastases and recurrence of SCLC, thereby attaining more durable benefits of chemotherapy and immunotherapy for SCLC patients.

Multiple preclinical and clinical studies of tumor immunity have revealed that the immune profile of the tumor microenvironment (TME) is crucial for tumor initiation and progression [6], suggesting that augmentation of T cell function in the TME has therapeutic promise [7]. Previous studies reported that high levels of tumor-infiltrated T cells (TIICs) were associated with good prognosis for patients with many solid cancers [8, 9], and also closely correlated with good responses to traditional chemotherapy and/or radiotherapy [10, 11], indicating that TIICs have vital effects on prognosis and therapeutic response for cancer patients.

Immunotherapy could be an effective measure to further improve their long-term outcomes causing the limited therapeutic options for SCLC patients. Although immune checkpoint inhibitors (ICIs) have become an established therapeutic option in lung cancer therapy [12], the absolute prolonged benefit of ICIs is moderate, and only a small fraction of SCLC patients respond to immunotherapy [13]. Therefore, a deeper understanding of immune system function and the effects of immunosuppression in the TME may provide insight into new methods of broadening the clinical benefits of immunotherapy [14]. In the TME, T cells are the primary effectors against cancer cells, and T cell activation requires two signals. The first signal leading to T cell activation is an antigen-specific signal mediated by binding of the T cell receptor (TCR) to an antigenic peptide complexed with the major histocompatibility complex (MHC). However, TCR engagement is not sufficient for initiation of full T cell activation, and additional costimulatory signals expressed on the surface of antigen-presenting cells (APCs) are necessary to initiate maximum stimulation [15]. The integration of costimulatory/coinhibitory molecules could result in T cell survival, proliferation, and functional differentiation [16].

Considering the essential role of costimulatory molecules in T cell activation, we explored the profiles and clinical relevance of such molecules in SCLC. Several well-described costimulatory molecules (PD-1/PD-L1, CD86/CTLA4) of the B7-CD28 superfamily play an important role in regulating T cell activation [17, 18]. B7 family ligands are peripheral membrane proteins that are expressed on APCs. The activation of different combinations of CD28 family receptors on T cells can augment or attenuate immune responses by inducing costimulatory or coinhibitory signals [19]. Members of the B7-CD28 superfamily include 13 molecules (CD80, CD86, PD-L1, PD-L2, B7-H3, B7x, ICOSLG, and HHLA2) from the B7 family and 5 molecules (CD28, CTLA4, PD-1, ICOS, and TMIGD2) from the CD28 family. The tumor necrosis factor (TNF) family includes 19 ligands and 29 related receptors, which play a pivotal role in survival, proliferation, and differentiation of cells, including immune and non-immune cells [20]. These costimulatory molecules, consisting members of the B7-CD28 and TNFSF/TNFRSF families, constitute a comprehensive set of potential targets for novel immunotherapeutic agents. However, the expression profiles and clinical significance of these molecules in SCLC remains largely known.

Our study aimed to comprehensively uncover the molecular features and clinical relevance of costimulatory molecules in SCLC, which led us to develop a novel costimulatory molecule-based signature (CMS) as a predictor for estimating clinical outcomes and predicting chemotherapy response in SCLC patients with robust predictive power. The accuracy of this classifier as a prognostic marker was confirmed by assessing its predictive performance in different years and various clinical factors via receiver–operator characteristic (ROC) and concordance index (C-index) analysis. The CMS identified in this study was validated using qPCR data for 131 formalin-fixed paraffin-embedded (FFPE) specimens from the National Cancer Center (NCC). Moreover, the relationship between the CMS and the immune landscape, as well as the relationship between the CMS and immune checkpoints, were further explored. Our findings demonstrate that the CMS reported in this study can be used for prognostic stratification and identification of effective treatment options, thereby offering renewed hope for patients with SCLC.

## Materials and methods

### Public datasets

The original gene expression data for 110 SCLC patients were downloaded from Cbioportal (https://www.cbioportal.org/study/summary?id=sclc_ucologne_2015) and used as the training cohort in our study. All patients had complete clinicopathological factor data and overall survival data at the 5-year follow-up assessment. The Gene Expression Omnibus (GEO) (https://www.ncbi.nlm.nih.gov/geo/) is a public database that provides free access to high-throughput genomic data. We acquired microarray data from the GEO for an analysis of the distribution of costimulatory molecules between normal and SCLC tissues (accession number GSE40275).

### Patients and tissue samples

From January 2009 to November 2018, 131 NCC patients with an initial clinical diagnosis of SCLC were enrolled in the study. Specimens were collected by surgical resection under a strict standard operating procedure and archived in a FFPE tissue block. Detailed patients and tumor characteristics are summarized in Supplementary Table 1. The study was approved by the Ethics Committee of the Cancer Hospital of the Chinese Academy of Medical Sciences, and informed consent was obtained from all patients participating in this study.

### RNA extraction and quantitative real-time polymerase chain reaction (qRT-PCR)

Total RNA was isolated from FFPE SCLC tissues using RNAiso Plus reagent (Takara, #9109) according to the manufacturer’s instructions. Samples with optical density (OD) A260/A280 ≥ 1.8 and OD A260/A230 ≥ 2.0 were further detected by qPCR. cDNA for qRT-PCR was synthesized using the Prime Script™ RT reagent kit (Takara, #RR047A). The PCR reaction was performed using SYBR Premix Ex Taq II (Takara, #RR820A) in a final reaction volume of 10 μL, which included 1 μL template, 1 μL of each PCR primer, 3 μL nuclease-free water, and 5 μL SYBR Green Master Mix (Invitrogen). Each sample was assessed in triplicate, and expression values were normalized to that of GAPDH. All data were log2 transformed and analyzed using Agilent Mx3005 qRT-PCR system. The stable ΔCt values of GAPDH indicate that the results are reliable. Primers for the selected genes were designed using FastPCR software (http://primerdigital.com/fastpcr.html) and using NCBI Nucleotide Blast (http://blast.ncbi.nlm.nih.gov/blast) to ensure that transcript variants of these markers would be amplified. More information regarding the primers is presented in Supplementary Table 2.

### Functional enrichment analyses

Functional enrichment analysis was implemented with Gene Ontology (GO) and Kyoto Encyclopedia of Genes and Genomes (KEGG) analyses in DAVID 6.8 (http://david.abcc.ncifcrf.gov), which is a powerful tool for classifying functionally related genes or associated biological terms into biological modules. The key terms from the GO analysis and KEGG pathways are presented in bar charts.

### Gene set enrichment analysis (GSEA)

GSEA (http://www.broadinstitute.org/gsea) is a powerful analytical tool that is used to interpret genome-wide RNA expression data to gain insight into cellular function [21]. The significantly enriched gene sets were determined by false discovery rate (FDR) Q-value, normalized enrichment score (NES), and *P* value.

### Gene set variation analysis (GSVA)

According to the gene expression level, we conducted GSVA to score the molecular characteristics and gene sets of individual samples [22]. The analysis was performed with the GSVA package for the R Programming Environment, which is freely available from http://www.bioconductor.org.

### TIIC profile assessment and microenvironment cell population (MCP) counting

The ESTIMATE algorithm was implemented to assess the levels of infiltrating immune and stromal cells, as well as to estimate tumor purity. The scores were calculated using the estimate package in R software. Besides, immune infiltration by eight types of immune cell and two kinds of stromal cells was estimated using the MCP-counter method as implemented in the R Programming Environment [23]. This analysis utilizes a novel computational method and allows for comparison of proportions and pathways associated with infiltrating cells.

### Statistical analysis

Univariate survival and the least shrinkage and selection operator (LASSO) Cox regression analysis was performed to filter and identify the costimulatory molecules that were most closely related to prognosis. Next, the CMS was built based on the selected molecules. Risk scores were calculated to classify individuals into high- and low-risk groups according to the optimal cutoff point. Survival analysis was performed using the Kaplan–Meier method to evaluate the prognostic significance of the CMS in different groups, and the Log-rank test was used to compare survival curves. To validate the prognostic ability of the CMS, we generated ROC curves and subsequently calculated the area under the ROC curve (AUC). C-index was also calculated to determine the discriminatory power of these models. To determine whether the CMS was an independent risk factor for clinical outcomes, univariate and multivariable Cox proportional hazards regression analysis was used to evaluate the association of the CMS with clinical outcomes, with adjustment for the factors of age, sex, SCLC staging, and tobacco use. The hazard ratio (HR) and its 95% confidence interval (CI) were obtained using the Cox regression model. The relationships between CMS and other immune checkpoints were assessed by Pearson’s correlation analysis. Statistical analysis was performed with R 3.5.1, and a *P* value < 0.05 was considered to be statistically significant.

## Results

### Landscape and genetic variation of costimulatory molecules in SCLC

**Fig. 1:**
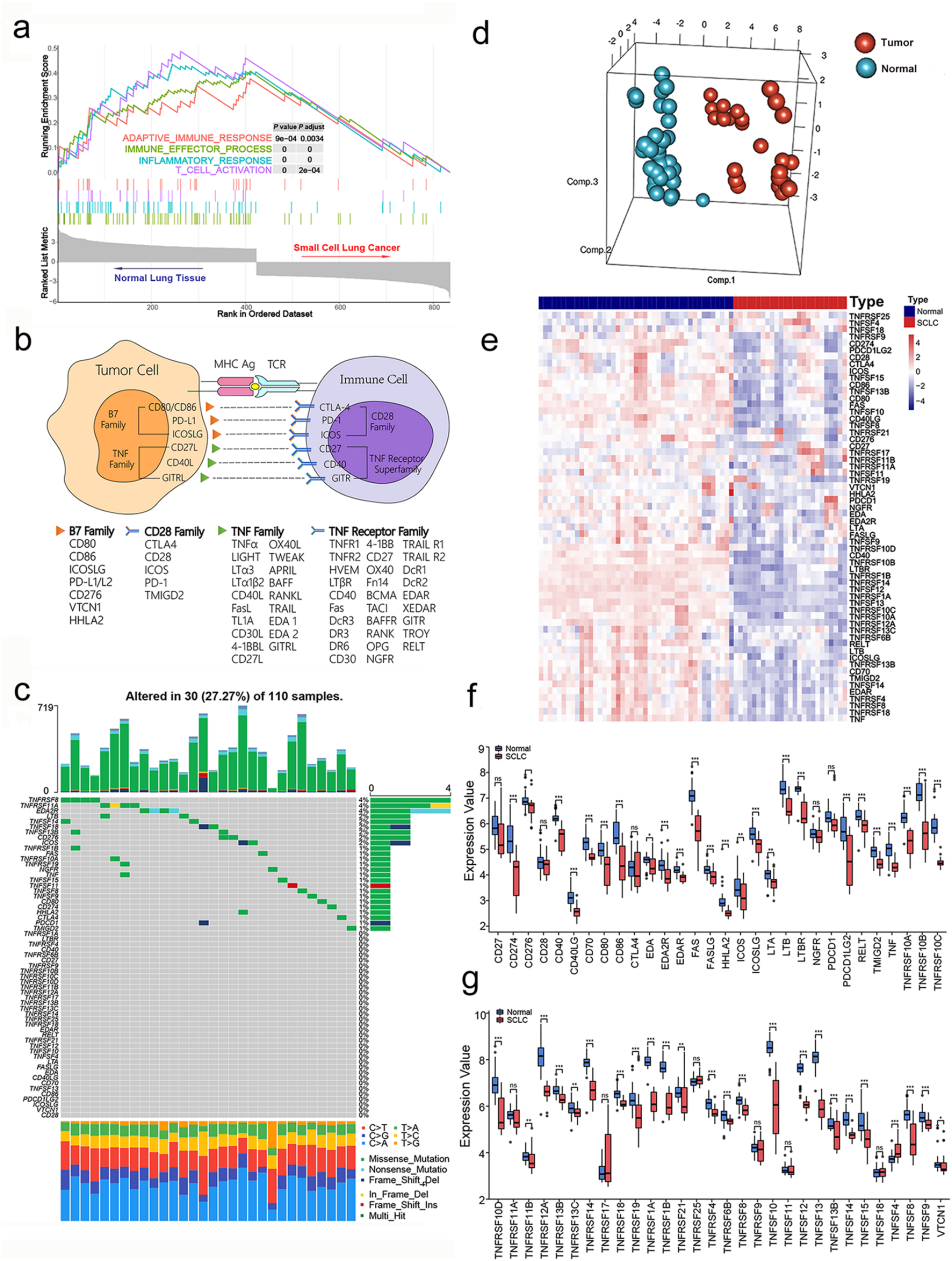
The molecular landscape of costimulatory molecules in patients with SCLC. **a** GSEA analysis of the RNA-seq data from normal and SCLC tissues (GSE40275). **b** The costimulatory molecules from B7-CD28 family and tumor necrosis factor superfamily (TNFSF) and TNF receptor superfamily (TNFRSF). **c** Somatic mutations of costimulatory molecules from 110 samples in the training set. **d** Principal component analysis of normal and SCLC tissues based on the expression profiles of costimulatory molecules (GSE40275). **e** Heatmap of the expression pattern of costimulatory molecules in samples of normal lung tissues and SCLC tissues (GSE40275). **f**–**g** The expression details of costimulatory molecules between normal and SCLC patients (GSE40275). *, **, and ***represent *P* < 0.05, *P* < 0.01, and *P* < 0.001, respectively. “ns” means not significant

GSEA analysis revealed that inhibition of T cell activation is a typical feature of SCLC (Fig. 1a). Considering the essential role of costimulatory molecules in regulating T cell response, we systematically explored the expression profiles of costimulatory molecules from B7-CD28 and TNF families. The B7-CD28 family consists of 8 ligands (CD80, CD86, ICOSLG, PD-L1/L2 CD276, VTCN1, and HHLA2) and 5 receptors (CTLA4, CD28, ICOS, PD-L1, and TMIGD2), and the TNF family consists of 19 TNFSF ligands and 29 TNFRSF receptors, which are identified in previous studies (Fig. 1b). Next, considering the essential role of costimulatory molecules in T cell-related immune responses, we visualized the characteristic mutation spectrum of those molecules in a water plot (Fig. 1c). Exploration of somatic mutations of 61 costimulatory molecules in 110 SCLC patients revealed that 30 samples (27.7%) showed mutations. Missense mutations were the most common type of mutation in the SCLC samples. We investigated the discriminatory capacity of costimulatory molecules in SCLC patients by using three-dimensional principal component analysis (PCA) to clearly differentiate groups based on gene expression levels (Fig. 1d). In addition, a heatmap showing the variance in the gene expression between normal lung and SCLC tissues was generated (Fig. 1e). Details information regarding the expression levels of costimulatory molecules is presented in Fig. 1f, g. A correlation matrix exploring the relationships between costimulatory molecules showed positive relationships with each other, as well as potentially important synergistic effects (Supplementary Fig. 1).

### Robust prognostic gene identification and CMS construction in the training cohort

**Fig. 2:**
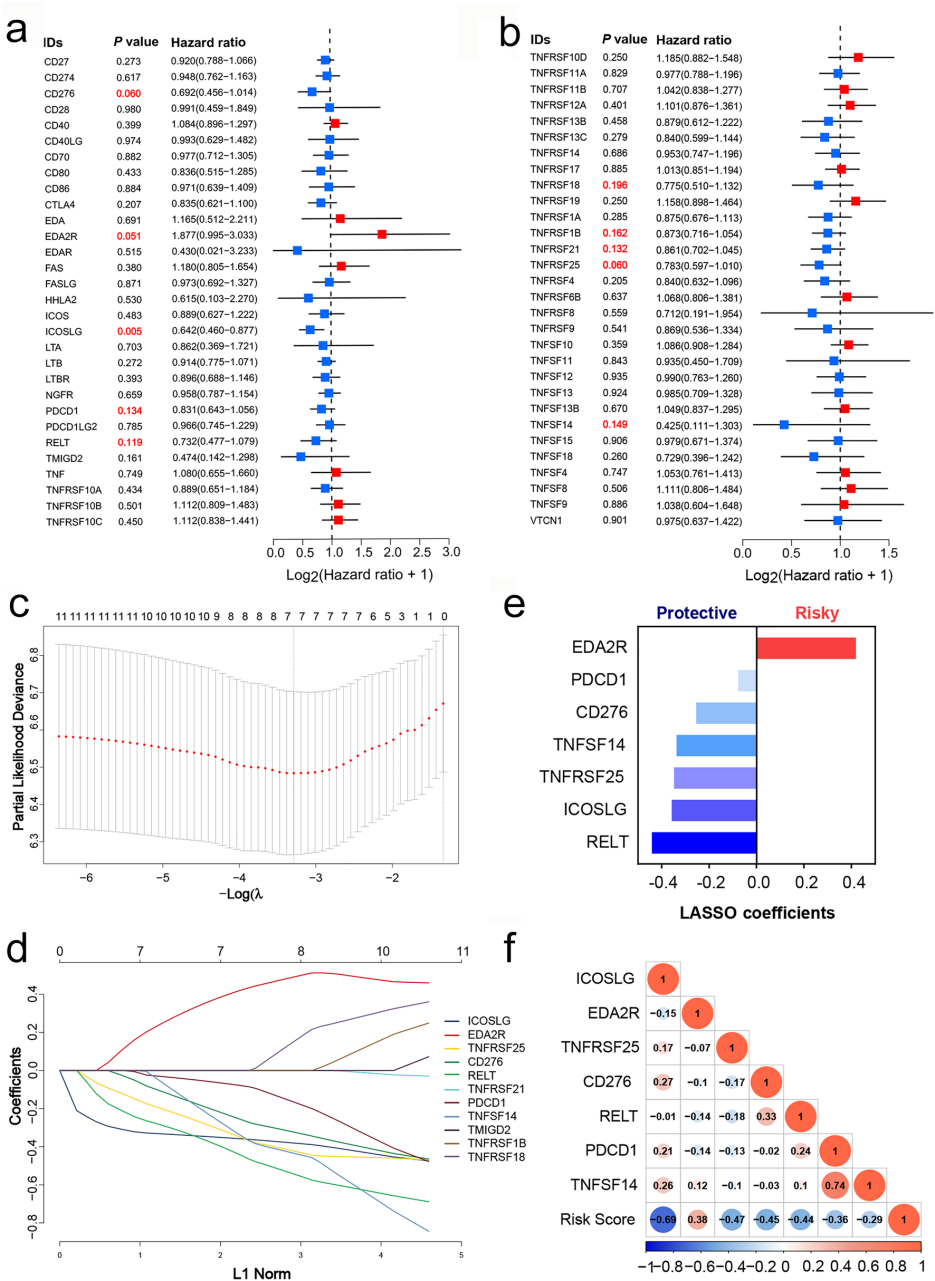
Filter out the most prognostic-related costimulatory molecules in patients with SCLC. **a**, **b** Forest plots displayed the univariate analysis results of costimulatory molecules in SCLCs. **c** Cross-validation error curve for tuning parameter (*λ*) selection in the LASSO model. **d** Lasso Cox coefficient profiles of costimulatory molecules. **e** Weighted LASSO Cox coefficients of selected prognostic genes. **f** Correlation matrix showed the association between the risk score and 7 selected genes of costimulatory molecules

**Fig. 3:**
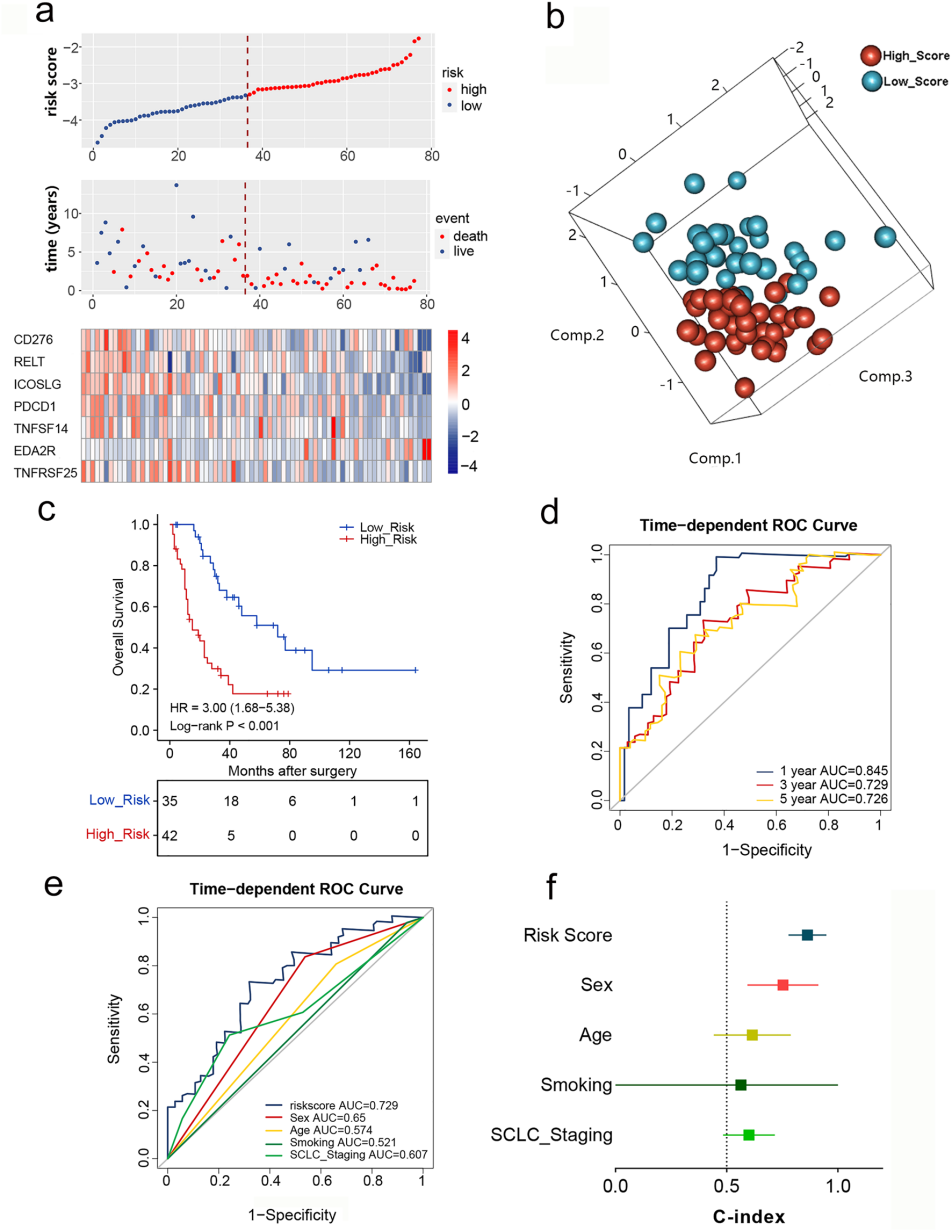
Generation of the costimulatory molecule-based signature (CMS) for patients with SCLC in the training cohort. **a** The risk score distribution, survival, and expression of costimulatory molecules. **b** Principal component analysis showed distinct difference between high- and low-risk patients based on the 7 selected genes of costimulatory molecules. **c** Kaplan–Meier curve for estimating OS based on CMS in patients with SCLC. **d** ROC curves for predicting OS at time points of 1, 3, and 5 years. **e** ROC curves for the performance of CMS and other clinical factors in predicting OS of SCLC patients. **f** C-index for CMS and other clinical factors in the training cohort

In order to develop a prognostic model for clinical use, we firstly used univariate Cox analysis to select valuable prognostic genes from the set of costimulatory molecules, which led to the selection of 18 genes with clinical relevance (*P* < 0.2) (Fig. 2a, b). Next, LASSO Cox regression was performed with seven prognosis-related genes, including six protective genes (PDCD1, CD276, TNFSF14, TNFRSF25, ICOSLG, and RELT) and one risk gene (EDA2R) (Fig. 2c–e). A set of 7 OS-relevant costimulatory molecules was used to build the CMS. The risk score formula was as follows: Risk score = (− 0.358 * ICOSLG) + (0.4192 * EDA2R) + (− 0.3482 * TNFRSF25) + (− 0.2544 * CD276) + (-0.4414 * RELT) + (− 0.0781* PDCD1) + (0.00671* TNFSF14). The risk score of each patient was calculated, and Pearson correlation analysis showed the relationship between risk score and the selected prognostic genes (Fig. 2f). Patients were ranked according to the continuous risk score and divided into high- (*n* = 42) and low-risk groups (*n* = 35) according to the optimal cutoff point (Fig. 3a). The 7 selected genes were differentially expressed between cases stratified by risk scores, and the PCA also showed significant heterogeneity between the high-risk and low-risk groups (Fig. 3a, b). Kaplan–Meier curves were applied to compare the OS of SCLC patients, and the results of this analysis indicated that the high-risk group had remarkably worse OS in comparison with that of the low-risk group (HR = 3.00, 95% CI: 1.68–5.38, *P* < 0.001) in the training sets (Fig. 3c). The prognostic accuracy of the CMS was determined using time-dependent ROC analysis at 1-, 3-, and 5-year follow-up assessments (Fig. 3d). Additionally, the performance of the CMS was compared to widely accepted prognostic and predictive factors for SCLC, including sex, age, tobacco use, and SCLC staging, using ROC curves (Fig. 3e). The AUC value of the risk score was highest for predicting survival (0.729). The risk score model achieved a C-index as high as 0.815 (Fig. 3f), suggesting that the CMS possesses good predictive capacity.

### Validation of the performance of the CMS for predicting clinical outcomes in the validation cohort

**Fig. 4:**
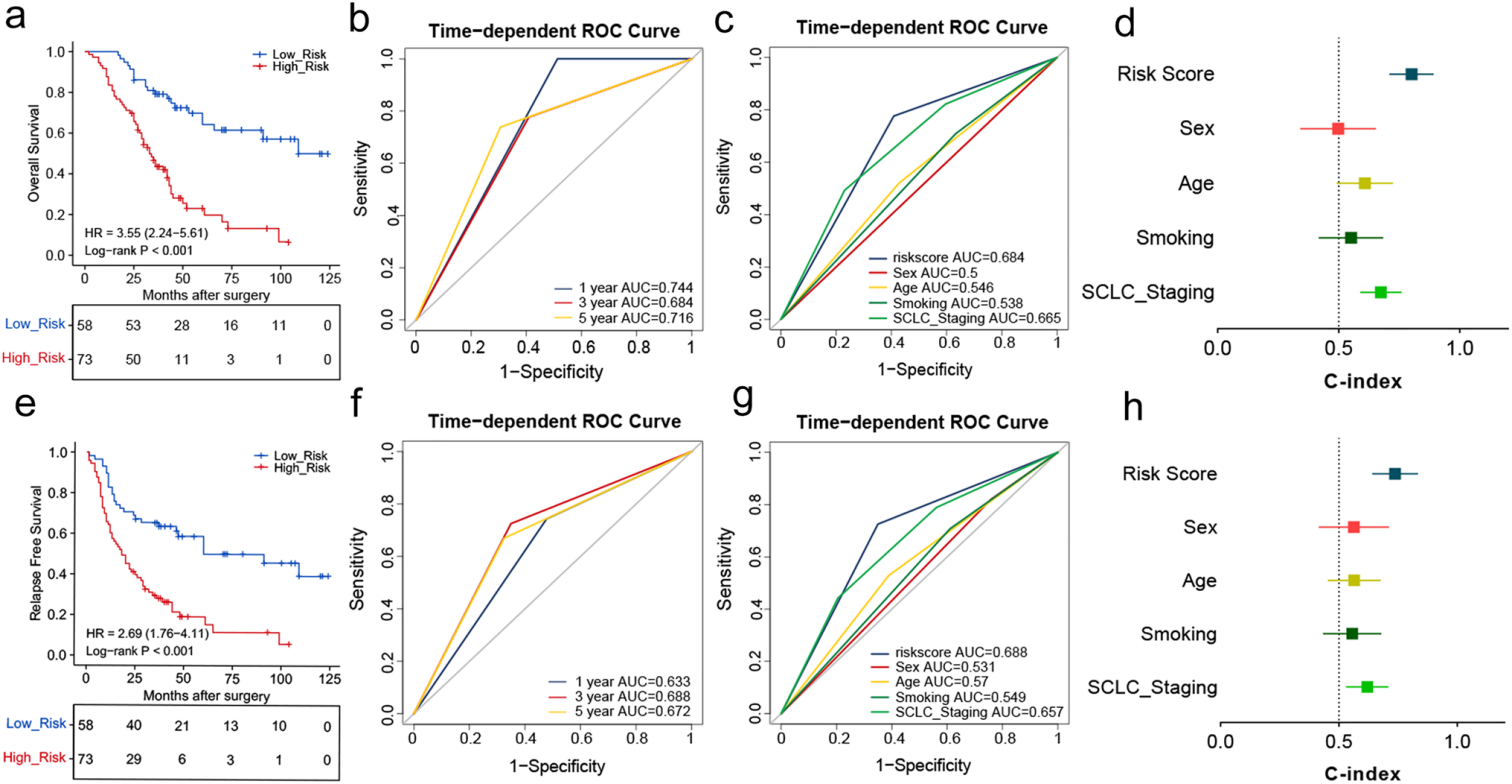
Validation of the prognostic performance of CMS for SCLC patients in the independent validation cohort. **a**, **e** The Kaplan–Meier curves for estimating overall survival (OS) and relapse-free survival (RFS) by the low- and high-risk groups. **b**, **f** ROC curves for predicting OS and RFS at time points of 1, 3, and 5 years. **c**, **g** ROC analysis of CMS compared with widely accepted predictive factors (age, sex, smoking, and SCLC staging). **d**, **h** C-index of models to predicting OS and RFS, respectively

To assess the prognostic ability of the CMS for SCLC in different population, we selected 131 SCLC FFPE samples with qRT-PCR data from the NCC as the validation cohort, and we calculated the risk score of each case. According to Kaplan–Meier analysis, we found that the high- (*n* = 73) and low-risk patients (*n* = 58) had significantly different survival status, and the high-risk group had worse outcomes (HR = 3.55, 95% CI: 2.24–5.61, *P* < 0.001) (Fig. 4a). We conducted a time-dependent ROC analysis to assess the prognostic accuracy of the CMS, and the AUC values at time points of 1, 3, and 5 years were 0.744, 0.684 and 0.716, respectively (Fig. 4b). A comparison of the predictive performance of the CMS (AUC = 0.684, C-index = 0.801) with other recognized factors, including sex (AUC = 0.50, C-index = 0.498), age (AUC = 0.546, C-index = 0.608), tobacco use (AUC = 0.538, C-index = 0.551), and SCLC staging (AUC = 0.665, C-index = 0.675), showed that the predictive accuracy of the CMS at 3-year survival was significantly better through the ROC and C-index analyses (Fig. 4c, d). Moreover, high- (*n* = 73) and low-risk patients (*n* = 58) showed an obvious difference in relapse-free survival (RFS) according to Fig. 4e (HR = 2.69, 95% CI: 1.76–4.11, *P* < 0.001), and the AUC values at 1, 3, and 5 years were 0.633, 0.688, and 0.672, respectively (Fig. 4f). Subsequent ROC and C-index analyses revealed that the CMS had better predictive capacity for RFS in comparison with the other predictive factors (Fig. 4g, h).

### Validation of the CMS in different clinical subgroups

Patients with complete clinical information were subjected to subgroup analysis to confirm the stability of the CMS in the training cohort. The subgroups included sex (male or female), age (age ≥ 60 or age < 60), and smoking history (smoker or non-smoker), The Kaplan–Meier analysis showed that high- and low-risk groups had distinct survivorship, and the low-risk group had obviously better OS among different subgroups (Supplementary Fig. 2).

### CMS is an independent risk factor for the prognosis of SCLC patients

To further explore whether the CMS could act as an independent predictive factor for SCLC patients, univariate and multivariate Cox regression analyses were performed using the training cohort (Supplementary Table 3). The results of this analysis indicated that the CMS was an independent risk factor for SCLC patients’ survival, controlling for sex, age, smoking status, and SCLC staging (HR = 3.997, 95% CI: 2.078, 7.688, *P* < 0.001). Next, the CMS was validated in the testing set of 131 SCLC patients with qPCR data, and the results also proved the CMS was an independent risk factor for predicting OS (HR = 3.696, 95% CI: 2.132, 6.409, *P* < 0.001) and RFS (HR = 2.566, 95% CI: 1.572, 4.186, *P* < 0.001) when adjusting for confounders (age, sex, smoking, and SCLC staging).

### The predictive value in estimating clinical benefits of adjuvant chemotherapy (ACT) for SCLC patients

ACT is the standard of care for SCLC after surgery, but most patients may develop drug resistance and the clinical efficacy is far from satisfactory. Therefore, we analyzed the relationship between risk score and ACT response in SCLC patients. In the training cohort, we calculated the risk score based on the same formula used above, and we divided patients into high- (*n* = 24) and low-risk groups (*n* = 26) by the optimal cutoff point. Based on the Kaplan–Meier analysis, the treatment benefits of ACT were higher for patients with low-risk scores (HR = 2.33, 95% CI: 1.11–4.87, *P* = 0.011) (Supplementary Fig. 3a). The AUC of risk score at time points of 1, 3, and 5 years was 0.84, 0.649, and 0.683, respectively, suggesting that the CMS had good predictive accuracy (Supplementary Fig. 3b). Moreover, the ROC curves demonstrated the clinical usefulness of the CMS (AUC = 0.649), which was significantly better than that of several recognized predictive factors, including sex (AUC = 0.615), age (AUC = 0.527), tobacco use (AUC = 0.503), and SCLC staging (AUC = 0.693) (Supplementary Fig. 3c). The C-index also confirmed the better predictive ability of CMS (C-index = 0.727) compared with other models (Supplementary Fig. 3d). In the validation cohort, we also investigated the association between risk score and ACT response, with RFS and OS used as treatment outcomes. Kaplan–Meier curves indicated that high- (*n* = 63) and low-risk patients (*n* = 49) had significantly different OS (HR = 3.62, 95% CI: 2.20–5.96, *P* < 0.001) and RFS (HR = 2.59, 95%CI: 1.63–4.10, *P* < 0.001) (Supplementary Fig. 3e, i), and patients with high risk suffered from worse clinical outcomes after ACT. The predictive accuracy of the CMS was further confirmed by subsequent ROC and C-index analyses, which demonstrated the superior predictive value of the CMS in comparison with that of previously recognized predictive factors (Supplementary Fig. 3f–h and Fig. 3j, k). Besides that, we conducted univariate and multivariate analysis among patients with ACT to further ensure the independent role of CMS in predicting the efficacy of ACT, and the results showed the CMS risk score could well predict the efficacy of ACT among patients with SCLCs (Supplementary Table 4).

### Functional enrichment analysis of the CMS

**Fig. 5:**
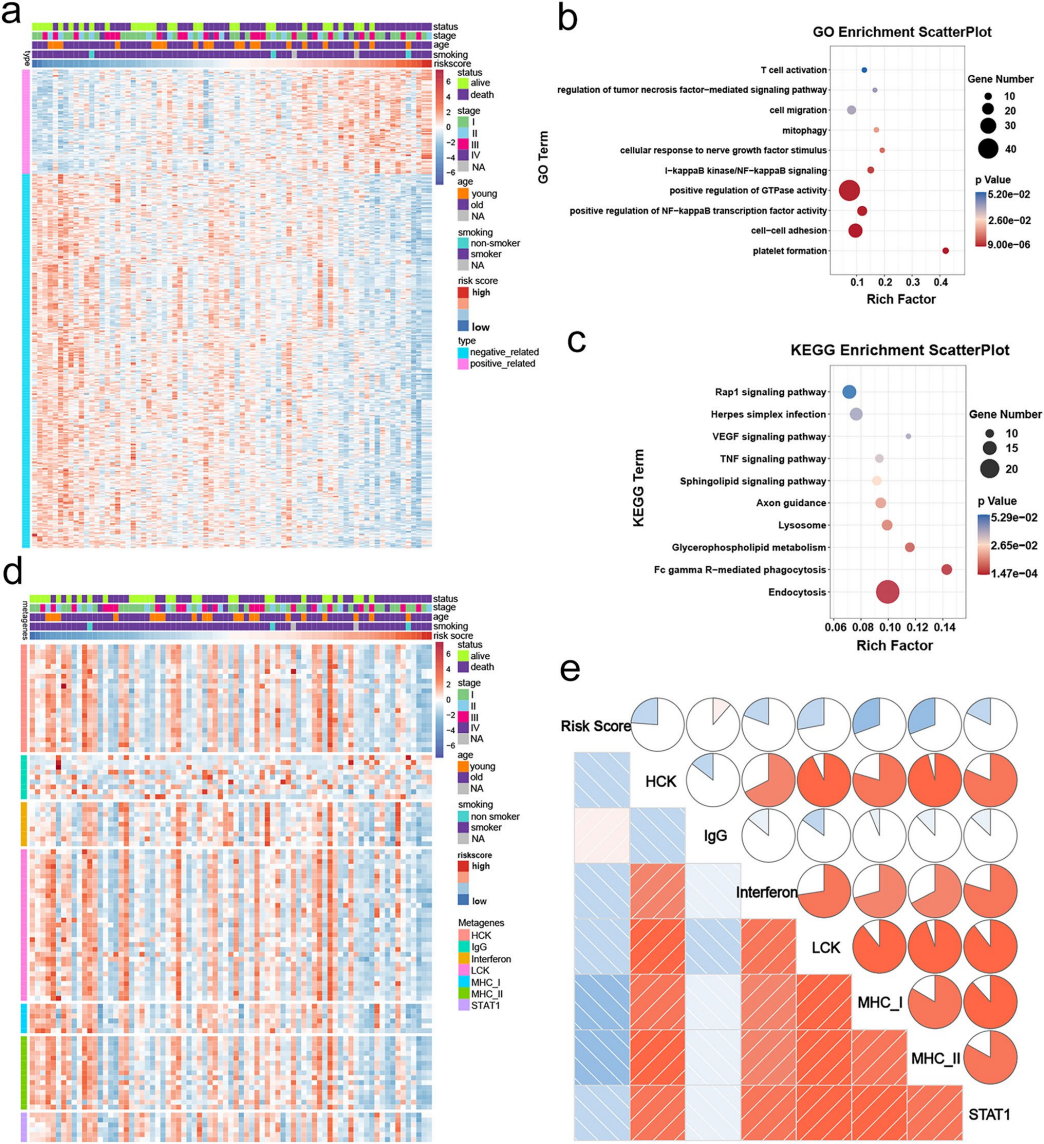
Biological pathways and inflammatory activities of CMS in the training cohort. **a** Heatmap of the CMS-related genes in SCLCs. **b**, **c** GO and KEGG analysis of these selected genes. **d** The expression profile of inflammatory metagenes and risk score in the training cohort. **e** The correlation between risk score and seven metagenes in the training cohort

The robustness of the CMS in predicting clinical outcomes prompted us to explore the biological role of the members of the CMS. We firstly filtered out genes that were closely related to the risk score (Pearson |R|> 0.4), and 670 negatively related genes and 187 positive related genes were selected (Fig. 5a). Next, we conducted GO and KEGG pathway analyses using the DAVID database. Our analysis revealed that the top enriched GO terms were related to immune response (Fig. 5b), and the KEGG analysis showed that the CMS was closely associated with immune-related pathways (Fig. 5c). Moreover, to better understand CMS-related inflammatory activity, the relationships between the CMS and seven metagenes were investigated. A metagene is a pattern of gene expression, and each metagene represents a cluster of genes that are functionally correlated. We generated seven metagenes using GSVA. The expression profile of the inflammatory metagenes and the risk score is presented in Fig. 5d. Pearson correlation analysis revealed that the risk score was negatively associated with some metagenes, including HCK, LCK, MHC_I, and MHC_II (Fig. 5e).

### TIICs and immune checkpoints associated with risk score

**Fig. 6:**
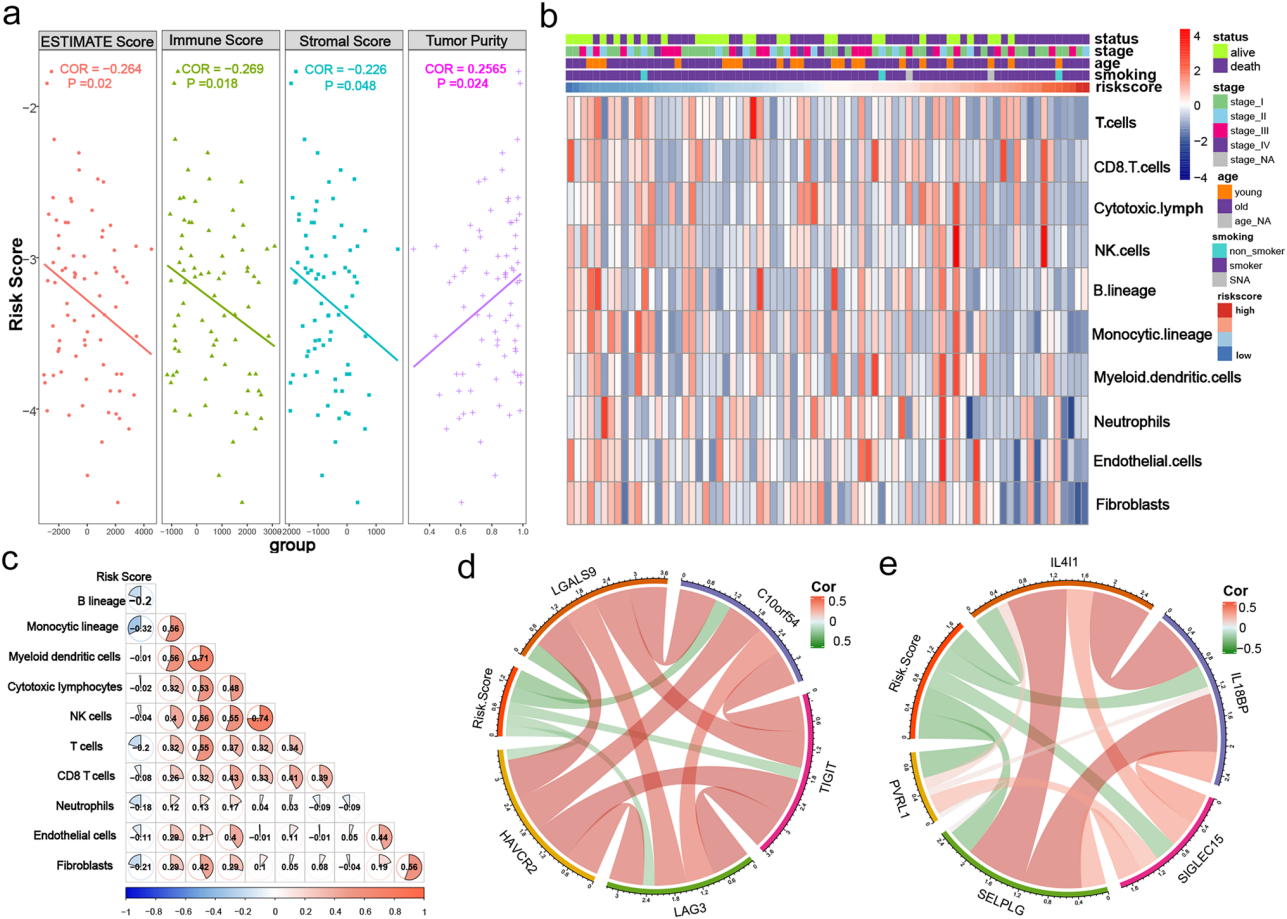
Relationship between risk score and infiltrating immune cells and immune checkpoints. **a** Association between risk score and immune infiltration status, including immune score, stromal score, and tumor purity. **b** Heatmap showing the immune cells landscape of SCLC patients with risk scores. **c** Correlogram showed the correlation between risk score and estimated immune cells. **d**, **e** Correlation between risk score and several novel immune checkpoints

Since TIICs are key elements of the TME that are essential for the carcinogenesis of SCLC, we characterized the expression patterns of immune cell infiltration in SCLC patients. ESTIMATE scoring was used to calculate the stromal and immune scores in the TME. The risk score was significant negatively correlated with the stromal score (Coefficient = –0.226, *P* = 0.018) and immune score (Coefficient =  − 0.269, *P* = 0.048), and positively correlated with tumor purity (Coefficient = 0.2565, *P* = 0.024) (Fig. 6a). The correlation matrix also showed a negative relationship between the risk score and various immune cells (Fig. 6b). The heatmap displayed distinct immune cell expression in high- and low-risk patients; low-risk patients showed greater infiltration by immune cells, especially CD8 + T cells (Fig. 6c). In addition, to examine the relationship between risk score and immune checkpoints in SCLC, Pearson correlation was performed and showed that risk score had negative concordance with other immune checkpoints in Fig. 6d, e.

## Discussion

SCLC remains one of the most lethal and aggressive malignancies, and its poor prognosis has remained consistent for decades. The rapid and remarkable advancement of high-throughput sequencing technology applications in cancer research has revolutionized our understanding of cancer biology, and the genomic features of tumors, as well as the immune status of the TME, have been recognized as valid biomarkers for predicting treatment response and prognosis [24, 25]. However, SCLC tumor specimens are extremely difficult to obtain under normal circumstances; therefore, the process of exploring prognostic prediction and treatment management based on tissue-specific biomarkers in larger-cohort studies has been slow and difficult. Therefore, developing a signature based on the intrinsic microenvironment of tumorigenesis to predict survival and clinical response of SCLC is a high research priority.

Costimulatory molecules, including the B7-CD28 superfamily and the TNFSF/TNFRSF family, are essential in modulating immune responses [16], and these molecules have well-established relationships with tumor prognosis and treatment response [26]. We first explore the differential pathways associated with the pathogenic processes of SCLC between tumor tissue and normal tissue. We found an immunosuppressive state, with reduced T cell-related inflammation, in SCLC patients, suggesting that T cell-associated tumor immune evasion may be the key mechanisms underlying SCLC development [27]. These results prompted us to systematically explore the molecular features and clinical potential of 61 costimulatory molecules in patients with SCLC. We constructed a first prognostic model based on the expression of seven costimulatory molecules in the training cohort. The identified signature was well-validated in the validation cohort of 131 patients from the NCC and performed well in different clinical subgroups. The predictive accuracy of the CMS was better than that of clinical predictive factors according to ROC and C-index analysis. The identified signature was found to be an independent prognostic factor in the multivariate analysis. Noticeably, the CMS had prognostic value in assessing the clinical response of ACT among SCLC patients. We also explored the immune profiles of SCLC patients through a bioinformatics approach, and we found that TIIC and inflammatory activity differed significantly between the high- and low-risk groups. Additionally, the CMS was negatively associated with other novel immune checkpoints. To the best of our knowledge, our study was the first to systematically characterize the immune profiles of SCLC patients and the clinical relevance of costimulatory molecules in such patients, and we identified a signature with significant prognostic and ACT response prediction value for patients with SCLC.

In order to conduct an intensive study of costimulatory molecules in SCLC patients, 61 recognized molecules from the B7-CD28 and TNFSF/TNFRSF families were assessed in our analysis. Using Kaplan–Meier survival analysis and LASSO Cox regression analyses, seven genes were identified. We found that most costimulatory molecules were protective factors, including CD276, ICOSLG, PDCD1, RELT, TNFSF14, and TNFRSF25, suggesting that higher expression levels of these molecules are closely associated with favorable prognosis. This finding confirms our previous conjecture that activation of costimulatory molecule-mediated signaling pathways is associated with long-term survival. Our previous study revealed the CMS could predict prognosis and immunotherapy response in lung adenocarcinoma [28], and we reevaluated the role of CMS in SCLC. We found the CMS could also well predict prognosis and chemotherapeutic response. The predictive signatures were constructed using different costimulatory molecules because of the distinct mechanism between different pathological types of lung cancer. The results further highlighted that the costimulatory molecules play a different and indispensable role in cancers with various clinical background and pathological classification.

CD276 (B7-H3) is a type I transmembrane protein and an independent costimulatory ligand of the B7-CD28 superfamily. Previous studies highlighted its therapeutic effect, and it has been recognized as an attractive target for cancer immunotherapy [29, 30]. PDCD1, also known as PD-1, is a member of the B7-CD28 family, and its ligands are PD-L1 and PD-L2. PDCD1 is mainly expressed on activated T cells, B cells, NK cells, and monocytes, and it regulates the activation and proliferation of these cell types [31, 32]. Previous studies identified PD-L1 as a prognostic factor, and its expression was associated with good prognosis for patients with various malignant tumors [33–35], including SCLC patients [36], which was consistent with our findings. ICOSLG, also known as B7-H2, is a cell-surface protein ligand that is mainly expressed on APCs and binds with the ICOS receptor to participate in regulating immune responses [37, 38]. The function of ICOSLG may be similar to that of PD-L1, and contributing to favorable prognosis for SCLC patients. RELT (TNFRSF19L) is a type I transmembrane glycoprotein that is abundant in immune cells and hematologic tissue [39]. Immobilized RELT has been shown to co‑stimulate T cell proliferation together with CD3 signaling, indicating a potential role in regulating immune responses [40]. TNFSF14, also known as LIGHT, is a member of the TNF ligand family. TNFSF14 binds with TNFRSF14 to deliver costimulatory signals to T cells that are capable of causing significant changes in the TME and induce an anti-tumor immune response, indicating that they might be useful in enhancing cancer immunotherapy [41, 42]. TNFRSF25 (DR3) is a type I membrane protein that binds to its ligand TNFSF15 (TL1A). The function of TNFRSF25 in cancer development and progression remains unclear [43]. However, in our study, we found that TNFRSF25 was associated with an overall poor prognosis in SCLC patients. In contrast with the other selected molecules, high expression of EDA2R was closely related to poor prognosis [44], suggesting that it could represent a bottleneck for immunotherapy for SCLC; future research should assess whether treatments targeting EDA2R can significantly enhance the efficacy of such immunotherapy.

The performance of the CMS was validated in an independent external cohort and different clinical subgroups. The identified signature had good predictive value as a method of stratifying patients into high- and low-risk groups with significant different clinical outcomes. In addition, the robustness of the model was confirmed in different clinical subgroups; although some of the *P* values indicated no statistical significance, there was still a tendency to separate the patients into high- and low-risk groups with different outcomes. In addition, the CMS demonstrated an advantage with regard to prognostic accuracy for chemotherapy resistance and survival over previously recognized predictive models based on sex, age, smoking status, and SCLC staging. Collectively, our findings demonstrated the important prognostic value of the CMS for patients with SCLC.

It is worth mentioning that the CMS also had predictive value in estimating the clinical response to chemotherapy for SCLC patients. We found that low-risk patients had favorable clinical outcomes compared with high-risk patients, suggesting that low-risk patients acquire more therapeutic benefit from chemotherapy. Numerous studies indicated that TIICs, as important components of the TME, have an essential role in host antigen-specific tumor immune responses and regulating therapeutic efficacy [11]. Our immune cell infiltration analysis revealed that the low-risk patients had highly abundant CD8 + TILs. This finding further highlighted the relationship between anti-tumor immune response and chemotherapy for SCLC patients. PD-L1 is a biomarker for chemotherapy in various cancers [45, 46], demonstrating that immune molecule-related biomarkers may be important guides for the application of chemotherapy in the future, and further indicating that the combination of immunotherapy and chemotherapy is expected to be a promising prospect for SCLC patients.

According to the above analysis, we confirmed that the CMS had good prognostic value, and our findings encouraged us to explore the underlying mechanism of the signature. We found that the CMS was closely associated with immune-specific biological processes. Considering the correlation between prognosis and immune pathways, the potential for immune-related pathways to determine prognosis was indirectly explained. The immune cell landscape was investigated, and the high- and low-risk groups were found to have different cell type compositions. Moreover, to better understand CMS-related inflammatory responses, the relationships between the CMS and metagenes were subsequently evaluated. A correlation chart revealed that the risk score was negatively associated with HCK, LCK, MHC_I, and MHC_II. MHC_II was found to be closely related to T cell presentation. Additionally, in order to investigate whether immunotherapy could improve therapeutic effectiveness for patients in different risk groups or act as an effective supplementary option for patients for which chemotherapy has failed or who show chemotherapy resistance, we assessed the relationship between the CMS risk score and other immune checkpoints. A negative relationship was observed between the risk score and immune checkpoints.

Thus far, clinically useful biomarkers to predict prognosis and treatment response in SCLC patients remain rare due to the paucity of tumor specimens, and existing predictive models lack clinical applications because they are limited by insufficient sample size. To our knowledge, our study utilized the largest sets of well-characterized samples and mature clinical data with long-term follow-up information, complete prognosis, and detailed clinicopathological information. The signature we identified has a favorable prognostic value for survival prediction and has the capacity to predict the clinical response to ACT for SCLC patients. The prognostic predictive power of the CMS was robust and stable in the training and validation cohorts, and its predictive accuracy was better than that of previously recognized risk factor-based models, providing a credible basis for its clinical utility.

However, some limitations of our study should be acknowledged. Our study had a retrospective design, and our sample size was limited; therefore, the predictive value of the CMS identified in our study should be further verified in a large prospective study. In addition, we used FFPE samples with limited quality and/or quantity in our study. Finally, the association between risk scores and the immune landscape was investigated by bioinformatics analysis; therefore, some noise was inevitable, and further biological experiments are required to validate our findings.

In conclusion, our study introduced a novel, predictive seven-gene signature (CMS) based on costimulatory molecules. This prognostic tool can effectively classify patients into different risk groups with distinct survival status, and its prognostic value is superior to that of traditional risk factor-based models. More importantly, it also possessed the capacity to predict the clinical response to ACT for SCLC patients, which should help clinicians to achieve personalized adjuvant therapy and prognostic management for SCLC patients.

### Supplementary Information

Below is the link to the electronic supplementary material.

Supplementary file1 (DOCX 2404 kb)

## Abbreviations

SCLC: Small cell lung cancer
TIICs: Tumor-infiltrated T cells
TME: Tumor microenvironment
ICIs: Immune checkpoint inhibitors
TCR: T cell receptor
MHC: Major histocompatibility complex
APCs: Antigen-presenting cells
TNF: Tumor necrosis factor
TIICs: Tumor-infiltrated immune cells
CMS: Costimulatory molecule-based signature
ROC: Receiver–operator characteristic
C-index: Concordance index
FFPE: Formalin-fixed paraffin-embedded
NCC: National Cancer Center
GEO: Gene Expression Omnibus
qRT-PCR: Real-time polymerase chain reaction
GO: Gene Ontology
KEGG: Kyoto Encyclopedia of Genes and Genomes
GSEA: Gene set enrichment analysis
GSVA: Gene set variation analysis
MCP: Microenvironment cell population
LASSO: The least shrinkage and selection operator
AUC: Area under the curve
HR: Hazard ratio
CI: Confidence interval
PCA: Principal component analysis
ACT: Adjuvant chemotherapy
