# Supplementary material for: Costimulatory molecule expression profile as a biomarker to predict prognosis and chemotherapy response for patients with small cell lung cancer
Source: Cancer Immunol Immunother. 2022 Aug 24;72(3):617–31. doi: 10.1007/s00262-022-03280-8 (PMC9947026; doi:10.1007/s00262-022-03280-8)
Supplement: Supplementary file 1 — Supplementary file1 (DOCX 2404 kb) [file 262_2022_3280_MOESM1_ESM.docx]

**Supplementary figures**

**Supplement Figure 1.** Correlation coefficient matrix of costimulatory molecules from B7-CD28 family and TNFSF/TNFRSF.

**
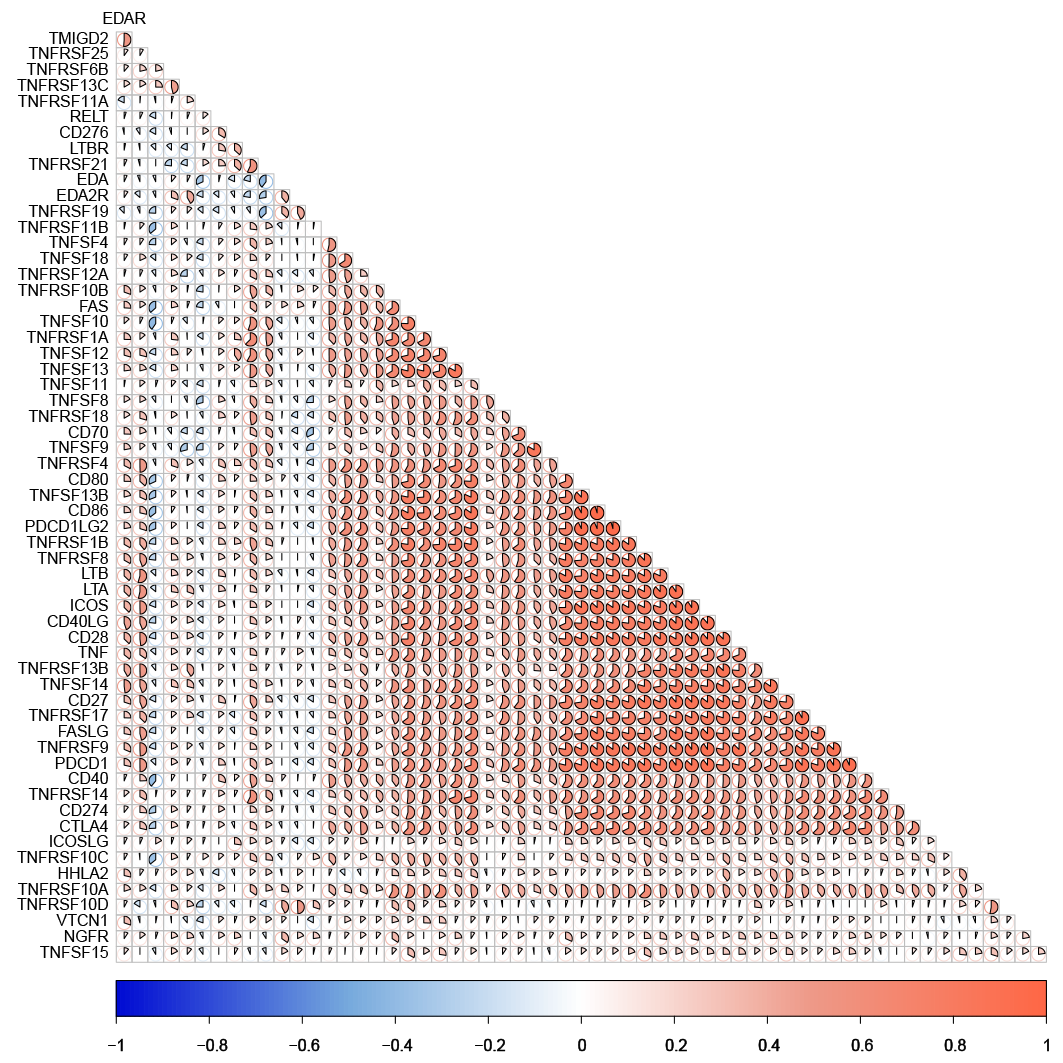
**

**Supplement Figure 2.** Kaplan-Meier curves for estimating OS and RFS of SCLC patients in clinical subgroups of the training and validation cohorts. K-M curves of OS in male (a), female (b), older (c), younger (d), and smokers (e) in the training cohort. K-M curves of OS in male (f), female (g), older (h), younger (i), smokers (j), and non-smoker (k) in the validation cohort. K-M curves of RFS in male (l), female (m), older (n), younger (o), smokers (p), and non-smoker (q) in the validation cohort.


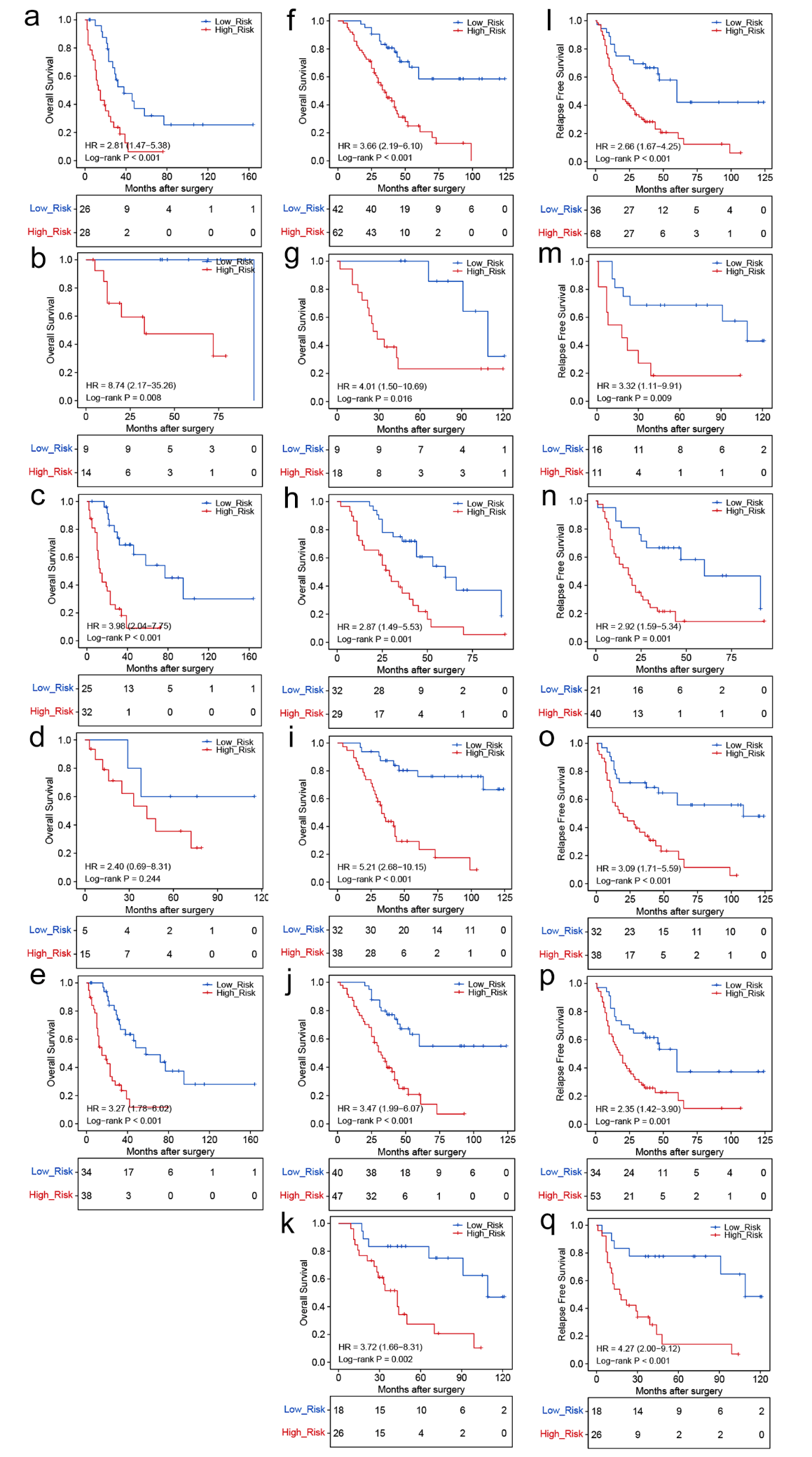


**Supplement Figure 3**. CMS predicts survival benefits for SCLC patients with adjuvant chemotherapy. (a) Kaplan-Meier curves of estimating OS based on CMS in the training cohort. (b) ROC curves for predicting OS at time points of 1, 3, and 5 years in the training cohort. (c) ROC curves for comparing the performance of risk score, sex, age, smoking, SCLC staging in predicting 3-year survival in the training cohort. (d) C-index for models to predicting OS in the training cohort. (e and i) Kaplan-Meier curves of OS and RFS based on CMS in the validation cohort, respectively. (f and j) ROC curves for predicting OS and RFS at time points of 1, 3, and 5 years in the validation cohort. (g and k) ROC curves for the performance of risk score, sex, age, smoking, SCLC staging in predicting 3-year OS and RFS in the validation cohort. (h and l) C-index for models to predicting OS and RFS in the validation cohort, respectively.


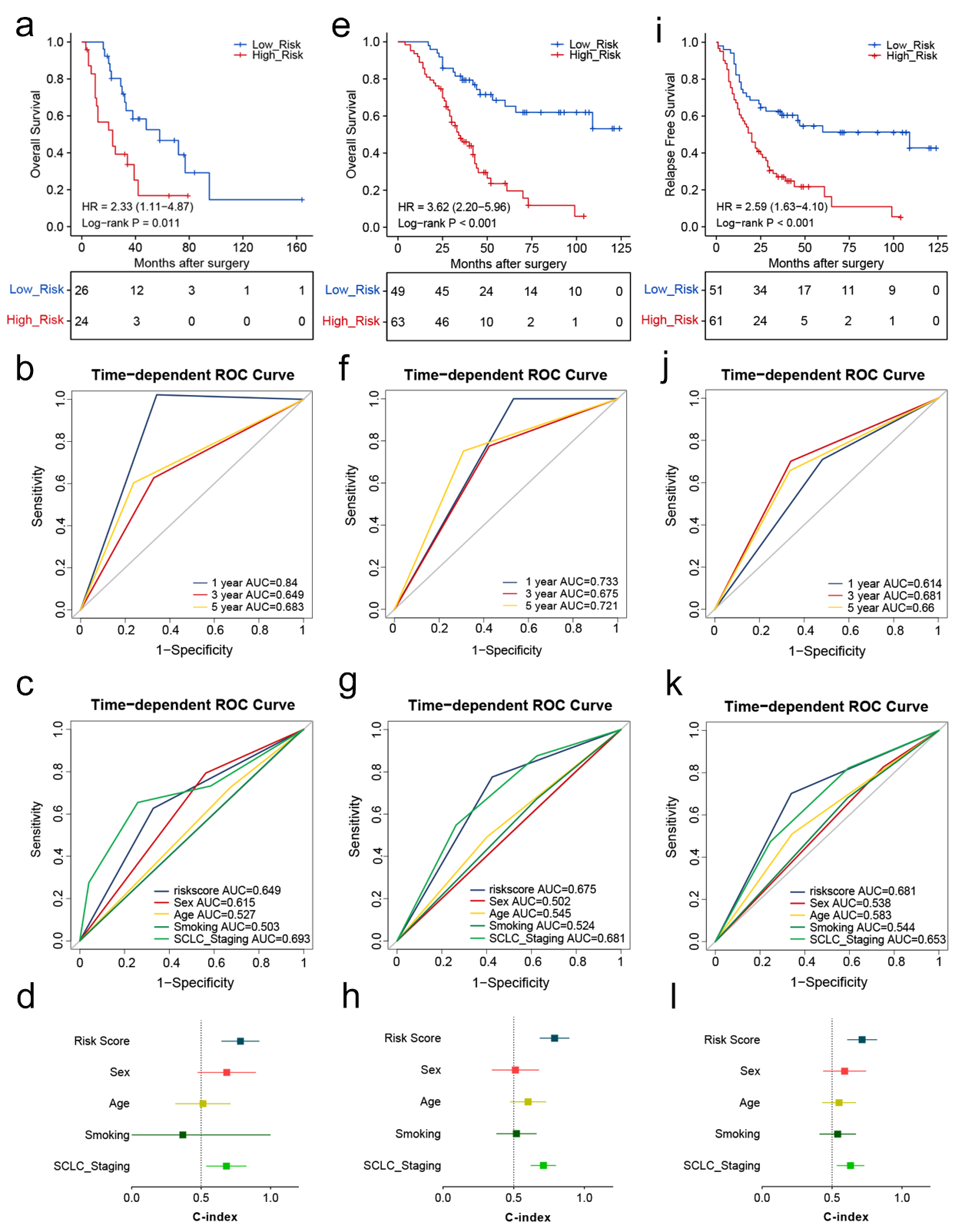


**Supplementary Table 1**. Clinical characteristics of patients enrolled in the training and validation cohorts.

| Characteristics | Training Cohort  (*N*=77) | Validation Cohort  (*N*=131) |
| --- | --- | --- |
| Sex |  |  |
| Female | 23 (29.87%) | 27 (20.61%) |
| Male | 54 (70.13%) | 104 (79.39%) |
| Age, years |  |  |
| <60 | 20 (25.97%) | 70 (53.44%) |
| ≥60 | 57 (74.03%) | 61 (46.56%) |
| Smoking history |  |  |
| No | 3 (4.00%) | 44 (33.59%) |
| Yes | 72 (96.00%) | 87 (66.41%) |
| SCLC staging |  |  |
| I | 33 (42.85%) | 41 (31.30%) |
| II | 14 (18.18%) | 46 (35.11%) |
| III | 21 (27.27%) | 44 (33.59%) |
| IV | 9 (11.69%) | 0 (0.00%) |
| OS state |  |  |
| Alive | 29 (37.66%) | 56 (42.75%) |
| Death | 48 (62.34%) | 75 (57.25 %) |

SCLC, small cell lung cancer; OS, overall survival.

**Supplementary Table 2**. Primer sequences of the samples used for qPCR in the validation cohort.

| **Gene Name** | **Forward Primer** | **Reverse Primer** |
| --- | --- | --- |
| CD276 | 5'-ACAGTTTCACCGAAGGCCG-3' | 5'-CTCAGGGATGCATTGCCTTGT-3' |
| ICOSLG | 5'-ATGTGGCAGCAAACTTCAGC-3' | 5'-TGATCCAGTACACGTTGGGC-3' |
| PDCD1 | 5'-TCGTGCTAAACTGGTACCGC-3' | 5'-CAGTTGTGTGACACGGAAGC-3' |
| RELT | 5'-TGGAGGCAGTGGAATCAACC-3' | 5'-TCTCTGTGATCAAGCGCACC-3' |
| TNFSF14 | 5'-CCGCTGTTATGGGAGACTCA-3' | 5'-CAGCTGCACCTTGGAGTAGA-3' |
| TNFRSF25 | 5'-CCCATCTGTCACCCTTGGAC-3' | 5'-TGTTACCCACCAACTGGACG-3' |
| EDA2R | 5'-GTTTTCCAGCTCTGCCTCCT-3' | 5'-ACATTGAGCTCCAGCCTGTC-3' |
| GAPDH | 5'-AAATCAAGTGGGGCGATGCT-3' | 5'-CAAATGAGCCCCAGCCTTCT-3' |

**Supplementary Table 3**. The univariate and multivariate analysis of prognostic factors in SCLC patients from different cohorts.

| Variable | Classification | Univariable analysis | | | Multivariable analysis | | |
| --- | --- | --- | --- | --- | --- | --- | --- |
|  |  | HR | 95% CI | *P* value | HR | 95% CI | *P* value |
| Training cohort (OS) |  |  |  |  |  |  |  |
| Risk score | High or Low | 3.322 | 1.790, 6.166 | <0.001 | 3.997 | 2.078, 7.688 | <0.001 |
| Sex | Male or Female | 2.948 | 1.374, 6.329 | 0.006 | 2.595 | 1.133, 5.947 | 0.024 |
| Age | ≥60 or＜60 | 1.493 | 0.759, 2.939 | 0.246 | 1.579 | 0.778, 3.205 | 0.206 |
| Smoking history | Yes or No | 2.437 | 0.334, 17.79 | 0.380 | 2.053 | 0.242, 17.426 | 0.510 |
| SCLC staging | IV, III, II, or I | 1.275 | 0.972, 1.673 | 0.080 | 1.481 | 1.105, 1.986 | 0.009 |
| Validation cohort (OS) |  |  |  |  |  |  |  |
| Risk score | High or Low | 3.920 | 2.317, 6.634 | <0.001 | 3.696 | 2.132, 6.409 | <0.001 |
| Sex | Male or Female | 1.114 | 0.637, 1.947 | 0.705 | 0.638 | 0.289, 1.41 | 0.267 |
| Age | ≥60 or＜60 | 1.733 | 1.088, 2.761 | 0.021 | 1.698 | 1.05, 2.745 | 0.031 |
| Smoking history | Yes or No | 1.204 | 0.739, 1.96 | 0.456 | 1.504 | 0.749, 3.019 | 0.251 |
| SCLC staging | III, II, or I | 1.637 | 1.216, 2.203 | 0.001 | 1.495 | 1.097, 2.037 | 0.011 |
| Validation cohort (RFS) |  |  |  |  |  |  |  |
| Risk score | High or Low | 2.882 | 1.808, 4.596 | <0.001 | 2.566 | 1.572, 4.186 | <0.001 |
| Sex | Male or Female | 1.443 | 0.833, 2.499 | 0.191 | 1.06 | 0.511, 2.202 | 0.875 |
| Age | ≥60 or＜60 | 1.352 | 0.879, 2.078 | 0.169 | 1.368 | 0.876, 2.137 | 0.168 |
| Smoking history | Yes or No | 1.288 | 0.816, 2.034 | 0.278 | 1.166 | 0.632, 2.151 | 0.624 |
| SCLC staging | III, II, or I | 1.493 | 1.134, 1.965 | 0.004 | 1.334 | 0.998, 1.782 | 0.052 |

HR, Hazard Ratio; CI, Confidence Interval; OS, overall survival; RFS, relapse-free survival.

**Supplementary table 4.** The univariate and multivariate analysis of prognostic factors in SCLC patients treated with ACT from different cohorts.

| Variable | Classification | Univariable analysis | | | Multivariable analysis | | |
| --- | --- | --- | --- | --- | --- | --- | --- |
|  |  | HR | 95% CI | *P* value | HR | 95% CI | *P* value |
| Training cohort (OS) |  |  |  |  |  |  |  |
| Risk score | High or Low | 2.500 | 1.213, 5.153 | 0.013 | 3.027 | 1.422, 6.443 | 0.004 |
| Sex | Male or Female | 2.343 | 1.005, 5.460 | 0.049 | 2.948 | 1.106, 7.862 | 0.031 |
| Age | ≥60 or＜60 | 0.967 | 0.460, 2.032 | 0.929 | 1.212 | 0.542, 2.715 | 0.639 |
| Smoking history | Yes or No | 1.082 | 0.146, 8.020 | 0.939 | 0.301 | 0.032, 2.860 | 0.296 |
| SCLC staging | IV, III, II, or I | 1.650 | 1.151, 2.365 | 0.006 | 1.970 | 1.315, 2.952 | 0.001 |
| Validation cohort (OS) |  |  |  |  |  |  |  |
| Risk score | High or Low | 3.881 | 2.193, 6.870 | <0.001 | 3.396 | 1.888, 6.107 | <0.001 |
| Sex | Male or Female | 1.147 | 0.621, 2.120 | 0.662 | 0.877 | 0.385, 1.998 | 0.754 |
| Age | ≥60 or＜60 | 1.744 | 1.046, 2.910 | 0.033 | 1.733 | 1.024, 2.935 | 0.041 |
| Smoking history | Yes or No | 1.085 | 0.646, 1.820 | 0.757 | 1.142 | 0.560, 2.329 | 0.715 |
| SCLC staging | III, II, or I | 1.831 | 1.310, 2.560 | <0.001 | 1.727 | 1.220, 2.446 | 0.002 |
| Validation cohort (RFS) |  |  |  |  |  |  |  |
| Risk score | High or Low | 2.770 | 1.659, 4.626 | <0.001 | 2.324 | 1.368, 3.950 | 0.002 |
| Sex | Male or Female | 1.491 | 0.815, 2.727 | 0.195 | 1.281 | 0.591, 2.777 | 0.531 |
| Age | ≥60 or＜60 | 1.352 | 0.844, 2.165 | 0.210 | 1.330 | 0.813, 2.175 | 0.256 |
| Smoking history | Yes or No | 1.226 | 0.751, 2.001 | 0.415 | 1.010 | 0.534, 1.912 | 0.974 |
| SCLC staging | III, II, or I | 1.619 | 1.191, 2.200 | 0.002 | 1.482 | 1.075, 2.043 | 0.016 |

HR, Hazard Ratio; CI, Confidence Interval; OS, overall survival; RFS, relapse-free survival; ACT, adjuvant chemotherapy.
